# Chewing Gum in the Larynx: Foreign Body Aspiration or Iatrogenic Artifact? Challenges in Determining the Cause of Death in a Road Traffic Accident Victim With Resuscitation Intervention

**DOI:** 10.7759/cureus.67085

**Published:** 2024-08-17

**Authors:** Pushwant S Mattu, Matthew M Orde

**Affiliations:** 1 Pathology and Laboratory Medicine, Vancouver General Hospital, Vancouver, CAN; 2 Forensic Pathology, Pathology and Laboratory Medicine, University of British Columbia, Vancouver, CAN

**Keywords:** airway foreign body, difficult airway management, autopsy case report, dilemma, trauma resuscitation, artifact

## Abstract

Identifying the cause of death in road traffic incidents and the contributing factors is crucial for forensic investigations, public health research, and epidemiological studies. In this case, the discovery of chewing gum in the larynx during an autopsy complicated the forensic diagnostic process and challenged the determination of the primary cause of death. Our case report details a 53-year-old male driver involved in a fatal road traffic accident. First responders found him unconscious and unresponsive. Despite resuscitative efforts, including endotracheal intubation, he could not be revived. The autopsy revealed multiple blunt force injuries from the collision and chewing gum in the larynx. The gum may have been aspirated while driving, potentially causing choking, severe coughing, or reflex cardiac arrest, which could have led to sudden incapacitation and the accident. Alternatively, the gum might have been overlooked during intubation, possibly pushing it deeper into the airway and creating an iatrogenic artifact. The cause of death was attributed to multiple blunt force injuries, specifically head trauma. However, the possibility of foreign body aspiration leading to the accident or the gum being an iatrogenic artifact cannot be ruled out. This case report highlights the potential impact of airway foreign bodies on road accidents and the risk of iatrogenic artifacts during resuscitation. It underscores the importance of thorough airway evaluation, prompt recognition of potential obstructions, and accurate documentation in prehospital settings to prevent worsening obstructions, misdiagnoses, delays in diagnosis, and complications in future cases.

## Introduction

Road traffic accidents are a leading cause of death and disability worldwide. Determining the cause and circumstances of death in these cases is crucial for forensic investigation, public health research, and epidemiological studies. Incomplete initial assessments can overlook injuries and diagnoses, leading to morbidity, mortality, and diagnostic errors. Forensic autopsies play a vital role in identifying causes of death and contributing factors in traffic accidents, detecting undiagnosed lesions in 10-47% of trauma-related hospital deaths [1].

Unusual findings during autopsies can complicate the determination of the primary cause of death, especially when the clinical history or examination is inconclusive. In this case report involving multiple fatal injuries, the precise cause of death was complicated by the presence of chewing gum in the larynx, which could be due to foreign body aspiration during driving or iatrogenic artifacts from resuscitation efforts.

Foreign body aspiration is a life-threatening condition commonly seen in children but less common in adults. It is often overlooked as a cause of airway obstruction. Clinical manifestations can range from asymptomatic to life-threatening emergencies. It typically affects the elderly or those with impaired neurological function, alcohol consumption, psychiatric conditions, diminished gag reflex, during sleep, or after trauma [2]. Diagnosing foreign body aspiration in unconscious trauma patients is particularly challenging due to vague or nonspecific clinical signs. It remains an important and often unrecognized complication in trauma patients, requiring high suspicion for early recognition and prompt action [3].

There is limited literature on foreign body aspiration in adult drivers, highlighting the need for this autopsy case report to raise awareness about potential missed airway foreign bodies in road accident patients. This case report examines the potential role of chewing gum aspiration in contributing to a fatal road traffic incident and addresses iatrogenic artifacts resulting from post-accident resuscitation interventions.

## Case presentation

A 53-year-old male with no significant medical history was involved in a single-vehicle motor incident while driving downhill on a freeway. His vehicle veered from side to side, rapidly gaining speed, before striking a rock wall and ending up in a forested area. The victim remained inside the vehicle until extricated and was found unconscious and unresponsive by the first responders. Despite resuscitation efforts, including intubation, he could not be revived. No clinical documentation mentioned chewing gum prior to the autopsy. Consequently, the death was reported to the coroner, and a medicolegal autopsy investigation was initiated.

The external examination revealed an overweight adult male with a BMI of 29 kg/m², showing signs of attempted resuscitation, including endotracheal intubation, chest defibrillator pads, and chest bruising. There were multiple surface grazes, bruises, and predominantly shallow skin tears on the head and body.

**Figure 1:**
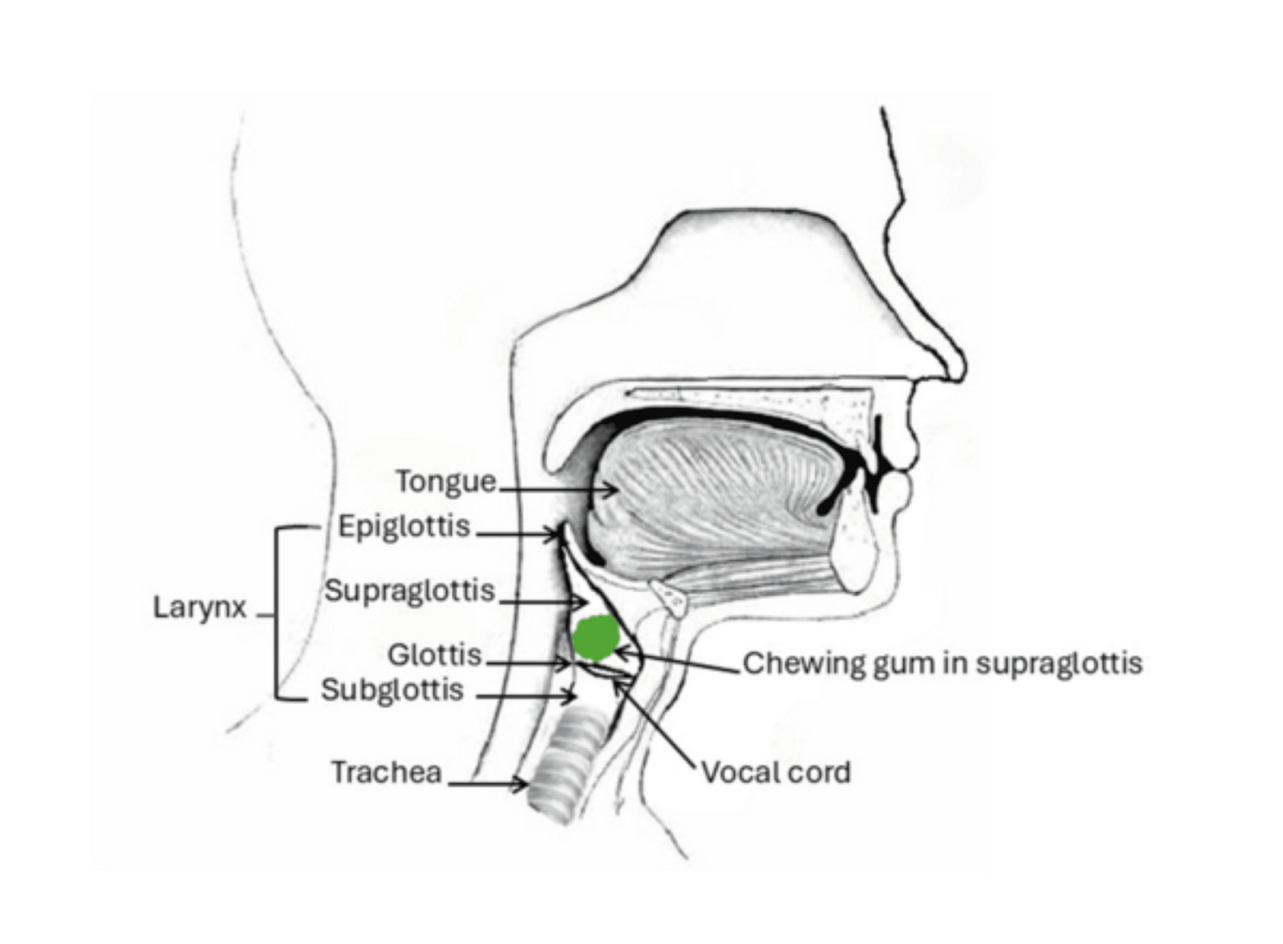
Green chewing gum found in the supraglottis of the larynx

The internal examination revealed bilateral anterolateral rib fractures and a transverse sternum fracture, with mild to moderate adjacent soft tissue hemorrhage, likely from resuscitation efforts. A green, malleable piece of chewing gum (15 × 8 × 7 mm) was found in the supraglottis of the larynx, accompanied by a minor smear of mildly hemorrhagic mucoid fluid (Figure 1). No intubation-related injury was observed in the pharyngeal, laryngeal, or tracheal mucosa. Airway mucosal surfaces showed a thin smear of hemorrhagic mucoid fluid, particularly in the distal bronchi. Lung histology indicated congestion with hemorrhage consistent with a contusion injury, along with excess mucus and minor intrapulmonary airway bleeding, without signs of acute bronchial asthma.

The heart was enlarged at 514 grams, showing left ventricular (LV) hypertrophy. The LV free wall thickness measured 18 mm, and the interventricular septum displayed asymmetric hypertrophy, particularly in the posterior region, measuring 23 mm. Mild coronary artery atherosclerosis was noted, with 20% stenosis in the proximal anterior descending artery and 10% in the right coronary artery. Microscopic analysis revealed nonspecific cardiac myocyte hypertrophy without significant disarray. Liver and kidney tissue samples were stored for potential genetic analysis, although this was not conducted.

**Figure 2:**
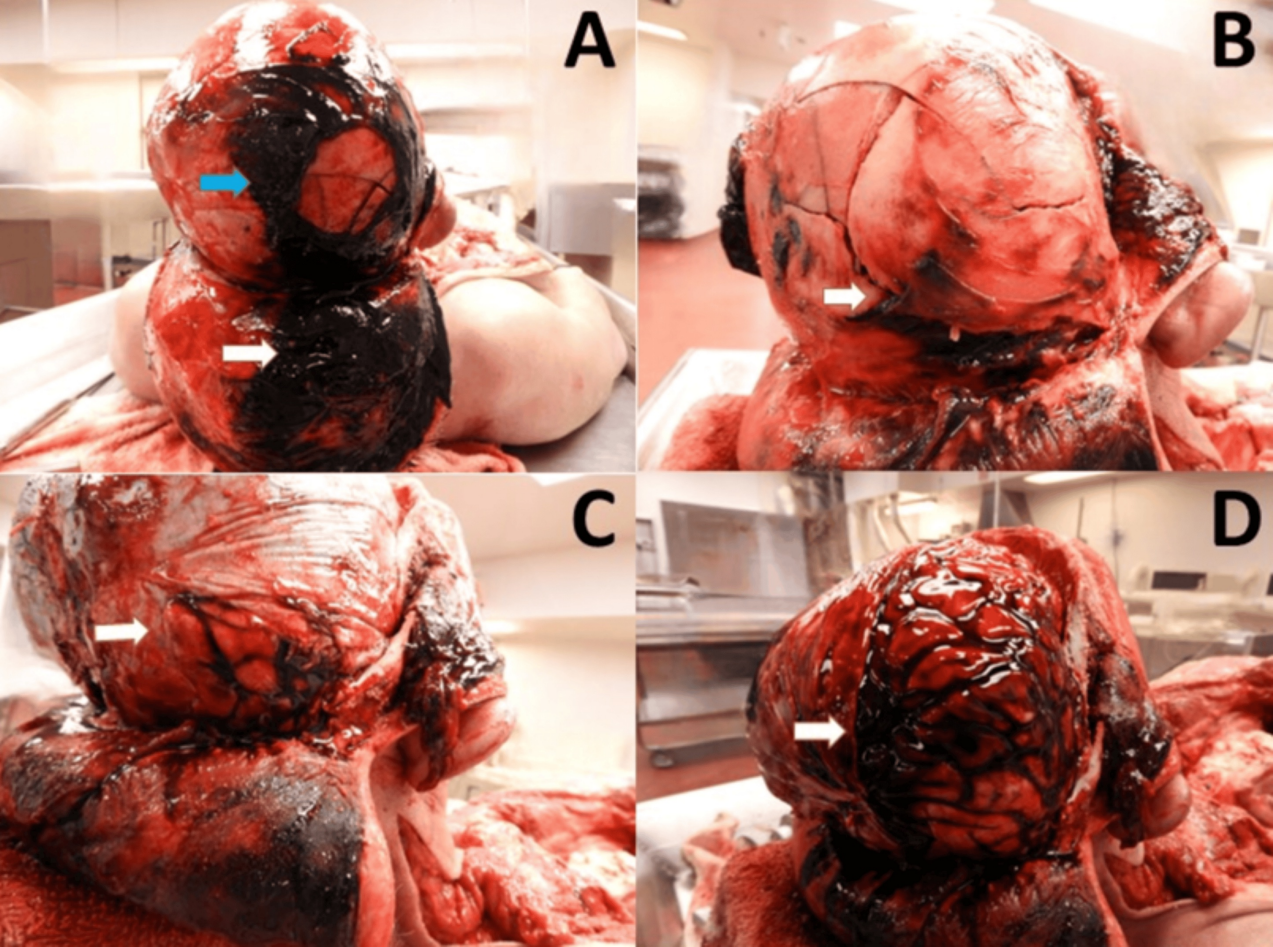
Autopsy findings of blunt head and brain trauma: scalp, skull, and cerebral injuries (A) Dense deep scalp bruising (blue arrow) and subaponeurotic hemorrhage (white arrow) in the right parieto-occipital region. (B) Complex fracture with a focal depressed component in the right parieto-occipital skull (white arrow). (C) Dural laceration in the right parieto-occipital region (white arrow) and diffuse, thin subdural hemorrhage. (D) Laceration in the right parieto-occipital cortex, underlying the area of maximum skull fracture, with multifocal subarachnoid hemorrhage and extensive cerebral contusion (white arrow).

Dense deep scalp bruising and subaponeurotic hemorrhage were observed, with a focal deep laceration in the right parieto-occipital region corresponding to external injuries. The undersurface of the scalp was otherwise essentially uninjured. A complex fracture (85 × 70 mm) was present on the right parieto-occipital skull, with a focal depressed component. Beneath it was a 48-mm dural laceration and bilateral acute, thin subdural hemorrhage, primarily located over the base of the supratentorial brain. A 92-mm ragged right parieto-occipital cerebral cortical laceration extended into the superficial white matter. Multifocal subarachnoid hemorrhage and extensive cerebral contusions were found, especially in the right temporal-parieto-occipital region, corresponding to the area of maximal skull injury (Figure 2). Additional injuries included a right frontal “gliding” contusion and a right dorsolateral brainstem hemorrhage. Microscopic analysis revealed hemorrhagic cerebral contusions, scattered white matter, and brainstem perivascular hemorrhages, with no underlying cerebral abnormalities.

Toxicology tests were negative for alcohol, drugs, and medications, and carbon monoxide levels were normal. However, the deceased sustained severe blunt force injuries, primarily to the head, which resulted in skull fractures and significant brain trauma. These injuries were determined to be the primary cause of death, with head trauma identified as the most significant contributing factor.

## Discussion

During the autopsy, the chewing gum lodged in the larynx was not photographed due to the predominance of severe head and brain injuries, which overshadowed any potential contribution of the gum to the road traffic accident. Following the autopsy, we explored the hypothesis that foreign body aspiration might have played a role in the accident, along with the possibility of iatrogenic artifacts arising from resuscitation interventions. This case report aims to highlight these considerations and raise awareness within the medical community about the potential impact of foreign body aspiration and resuscitation-related artifacts on road traffic accident cases.

The larynx is highly sensitive to mechanical or chemical stimuli, triggering protective reflexes against aspiration into the upper airway. These reflexes include retching, gagging, vomiting, sneezing, swallowing, coughing, and choking, involving vigorous neuromuscular activity and triggering respiratory, cardiovascular, and autonomic responses [4]. These mechanisms are mediated by the recurrent laryngeal nerve and the superior laryngeal nerve, with the latter primarily facilitating the laryngeal cough reflex. Aspiration of a small food particle can stimulate the larynx, inducing a violent cough, while mechanical and chemical irritants can also trigger coughing [5].

During a choking episode, a person may cough forcefully to clear the foreign object. However, if unsuccessful, this can lead to airway blockage and respiratory distress. The episode typically ends when the person expels the object by coughing it out, swallowing it, or spitting it out [2]. The outcome depends on the size and flexibility of the object. Large, sharp, or irregular objects can become lodged in the larynx [6]. Chewing gum’s size, flexibility, and movement in the larynx can lead to its upward and downward movement within the airway, causing laryngeal irritation [6] and potentially triggering a hyperreactive cough reflex, choking, laryngospasm, asphyxiation, or cardiac arrest.

In this case, the circumstantial data and postmortem findings suggest several potential pathophysiological mechanisms contributing to the road traffic incident. The first possible mechanism involves cough syncope, where prolonged severe coughing or choking induced by chewing gum aspiration into the larynx leads to unconsciousness and subsequent cardiac arrest [7]. This can occur due to increased intrathoracic and intraabdominal pressures, which reduce cardiac output and cerebral perfusion [8]. Another potential factor is cardiac arrest from vagal inhibition due to stimulation of the superior laryngeal nerve, possibly triggered by choking or irritation of the pharyngeal and laryngeal mucosa due to chewing gum [9].

A second possible mechanism suggests that severe choking from laryngeal aspiration of chewing gum may have caused fatal distraction [7], leading to loss of control while driving and resulting in a fatal accident with blunt injuries.

A third possible mechanism involves the significant cardiac enlargement found during the autopsy. Cardiomegaly, as observed, is associated with abnormal cardiac rhythms and spontaneous cardiac arrest, which could have contributed to the fatal accident. The specific cause of the heart disease remains unclear but may relate to preexisting hypertension or an underlying cardiomyopathic disorder [10].

A fourth possible mechanism involves the inhalation of a small foreign object that moves within the airway, potentially triggering the cough reflex and aiding in its expulsion. However, the victim’s inability to self-rescue and the absence of known risk factors for foreign body aspiration suggest that the victim may have been asleep before the choking episode, possibly while driving [6]. This scenario could have been exacerbated by sleep apnea due to a high BMI [11].

This motor vehicle crash shares similarities with incidents involving syncope, fatal distraction, and sleep-related factors, often resulting in collisions with stationary objects or veering off highways without braking at high speeds. These crashes are less influenced by factors such as road conditions, vehicle state, visibility, or weather [7].

However, despite the preceding discussion, it is acknowledged that the chewing gum may have moved from the mouth to the larynx either post-incident or during attempted resuscitation. It is possible that during endotracheal intubation, the gum was inadvertently pushed into the larynx, creating an iatrogenic artifact [12,13]. Chewing gum is often used preoperatively to alleviate dry mouth, reduce thirst, manage stress, and aid gastric motility. However, if the patient forgets to dispose of the gum before surgery, it may lead to complications, such as the gum being pushed deeper into the airway during intubation and adhering to the endotracheal tube post-intubation [14]. Forensic pathologists should be vigilant about these potential risks.

In this case, the cause of death is attributed to multiple blunt force injuries, particularly head trauma. However, the possibility that the vehicle accident resulted from sudden incapacitation due to chewing gum aspiration (e.g., choking, reflex vagal inhibition, or cough syncope) or that the gum’s presence in the larynx was an iatrogenic artifact cannot be ruled out. Diagnosing foreign body aspiration postmortem is challenging due to nonspecific signs in unconscious individuals. Additionally, the lack of patient history and clinical notes from airway assessment and intubation, along with diagnostic challenges, complicates the identification of a foreign body in the larynx as the causative agent. The literature on foreign body aspiration in adult drivers leading to traffic accidents is limited, and histological findings alone are inconclusive. Determining the role of reflex vagal stimulation during autopsy remains challenging [15].

Airway foreign body aspiration from chewing gum is rare in children [16], but has been reported in adults, leading to upper airway obstruction during events such as lethal asphyxiation [17], sudden cardiac arrest [18], sudden death [6], perioperative intubation obstruction under general anesthesia [14], and trauma or iatrogenic-induced aspiration with gum in their mouths [12,13].

In one study, choking events were correctly identified during CPR in only 5% of elderly individuals (aged 65+) and 13% of younger adults (aged 18-64). This lack of awareness is a crucial factor in foreign bodies being overlooked during airway management, which can compromise the effectiveness of resuscitation and intubation procedures. Such oversights can lead to irreversible brain damage or death within five to 10 minutes of the onset of mechanical obstruction. Furthermore, unawareness of a foreign body during intubation risks pushing it deeper into the throat and distal airway, potentially causing blockage and harm if the patient remains alive. Rapid recognition and immediate removal of obstructing materials during a thorough airway assessment before managing the airway could prevent these complications [19]. Literature reports a case where successful resuscitation occurred after cardiac arrest caused by airway obstruction from chewing gum, which was discovered in the larynx during rapid sequence intubation and removed to complete the procedure [18].

Hypoxia and airway mismanagement contribute to up to 34% of pre-hospital deaths in trauma patients. Therefore, suspecting airway obstruction in all trauma cases is crucial [3]. Key goals during resuscitation include securing an open airway and controlling the cervical spine. Managing the airway in life-threatening conditions is challenging, further complicated by factors such as mental stress, a large tongue, airway edema, potential neck injuries, immobilization, and the presence of vomit and blood [20]. Unrecognized foreign bodies, due to limited awareness and visibility, can render oxygen administration and mouth-to-mouth resuscitation ineffective until the blockage is cleared [19]. Proper airway assessment should include a thorough inspection for potential obstructions like shattered glass, plastic, or other debris in motor vehicle accident cases, as well as for secretions, bone fragments, or chewing gum. Ensuring safe intubation requires the removal of these foreign objects [12,13]. Reports indicated that in 125 out of 137 cases, the choking incident, which was the initial reason for emergency intervention, was not later identified as asphyxiation [19]. However, in urgent field intubations, a thorough assessment may not always be feasible. Patients arriving already intubated in the emergency department may falsely assume airway patency, potentially overlooking upper airway obstruction from foreign bodies [13].

Diagnosing foreign body aspiration in trauma and unconscious patients in prehospital settings is challenging but crucial. Many missed injuries or misdiagnoses could be prevented with vigilant clinical assessments and a high index of suspicion. Accurate documentation of injuries and examination findings, while assessing their plausibility in the context of the accident, is essential. This vigilance is particularly important during autopsies to accurately determine causes of death, such as choking or apnea, and to minimize potential iatrogenic artifacts.

## Conclusions

The unexpected finding of chewing gum in the larynx of a victim in a fatal road traffic accident complicates the determination of the cause of death. While blunt force injuries, primarily to the head, were identified as significant contributing factors, the presence of chewing gum introduces additional complexity to the forensic diagnosis. This case report explores how chewing gum aspiration might lead to fatal road accidents due to choking or reflex vagal inhibition while driving. It also considers the possibility of the gum being an iatrogenic artifact resulting from resuscitation interventions. Vigilance during airway assessment and intubation in prehospital settings is crucial to prevent misdiagnosis or delays in diagnosing airway foreign bodies. Accurate documentation of injuries and findings in prehospital settings is essential. Insights from this case report aim to improve the early recognition of foreign bodies, prompt airway intervention, and enhance documentation practices for trauma patients in future road accidents.
